# Supplementary material for: Cytotoxic Polyketide Metabolites from a Marine Mesophotic Zone Chalinidae Sponge-Associated Fungus Pleosporales sp. NBUF144
Source: Mar Drugs. 2021 Mar 26;19(4):186. doi: 10.3390/md19040186 (PMC8065988; doi:10.3390/md19040186)

Supplementary Information

Cytotoxic Polyketide Metabolites from a Marine Mesophotic Zone Chalinidae Sponge-Associated Fungus *Pleosporales* sp. NBUF144

Jing Zhou [1], Hairong Zhang [1], Jing Ye [2], Xingxin Wu [2], Weiyi Wang [3], Houwen Lin [4], Xiaojun Yan [1], J. Enrico H. Lazaro [5], Tingting Wang [1,*], C. Benjamin Naman [1], Shan He [1,*]

1 Li Dak Sum Marine Biopharmaceutical Research Center, Department of Marine Pharmacy, College of Food and Pharmaceutical Sciences, Ningbo University, Ningbo, Zhejiang, 315800, China
2 State Key Laboratory of Pharmaceutical Biotechnology, School of Life Sciences, Nanjing University, Nanjing, 210023, China
3 Key Laboratory of Marine Biogenetic Resources, Third Institute of Oceanography, Ministry of Natural Resources, Xiamen, 361005, China
4 Research Center for Marine Drugs, State Key Laboratory of Oncogene and Related Genes, Department of Pharmacy, Ren Ji Hospital, School of Medicine, Shanghai Jiao Tong University, Shanghai 200127, China
5 National Institute of Molecular Biology and Biotechnology, University of the Philippines Diliman, Quezon, 1101, Philippines
* Correspondence: wangtingting1@nbu.edu.cn (T.W.); heshan@nbu.edu.cn (S.H.)

Contents

Figure S1. LC–MS/MS derived molecular network of organic extracts produced from 80 fungal cultures (40 from shallow reef sponges, 40 from mesophotic zone sponges). Single node clusters, or self-loop nodes, were excluded for brevity. 2
Figure S2 ^{1}H NMR spectrum of compound **1** (600 MHz, $CDCl_3$) 3
Figure S3 ^{13}C NMR spectrum of compound **1** (150 MHz, $CDCl_3$) 4
Figure S4 DEPT135 spectrum of compound **1** (150 MHz, $CDCl_3$) 5
Figure S5 ^{1}H-^{1}H COSY spectrum of compound **1** 6
Figure S6 HSQC spectrum of compound **1** 7
Figure S7 HMBC spectrum of compound **1** 8
Figure S8 NOESY spectrum of compound **1** 9
Figure S9 ^{1}H NMR spectrum of compound **2** (600 MHz, $CDCl_3$) 11
Figure S10 ^{13}C NMR spectrum of compound **2** (150 MHz, $CDCl_3$) 12
Figure S11 DEPT135 spectrum of compound **2** (150 MHz, $CDCl_3$) 13
Figure S12 ^{1}H-^{1}H COSY spectrum of compound **2** 14
Figure S13 HSQC spectrum of compound **2** 15
Figure S14 HMBC spectrum of compound **2** 16
Figure S15 NOESY spectrum of compound **2** 17

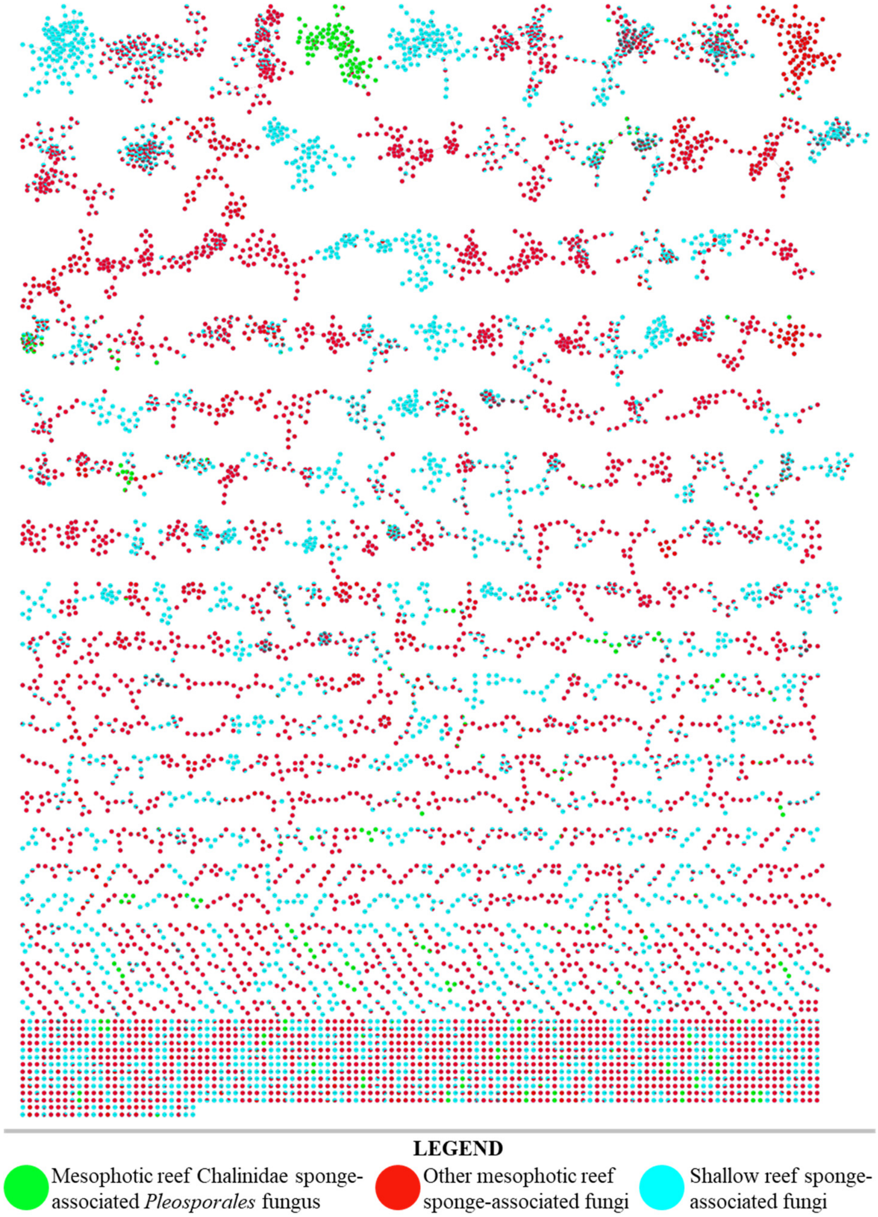

Figure S1. LC–MS/MS derived molecular network of organic extracts produced from 80 fungal cultures (40 from shallow reef sponges, 40 from mesophotic zone sponges). Single node clusters, or self-loop nodes, were excluded for brevity.

Figure S2 ^{1}H NMR spectrum of compound **1** (600 MHz, $CDCl_3$)

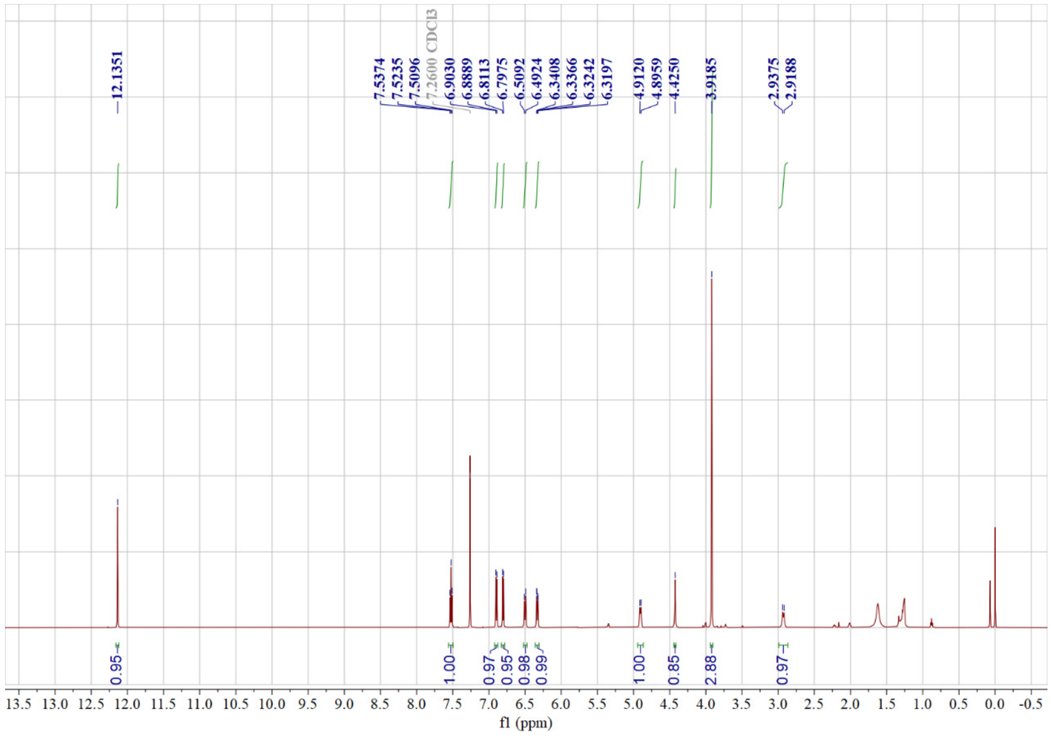

Figure S3 ¹³C NMR spectrum of compound **1** (150 MHz, $CDCl_3$)

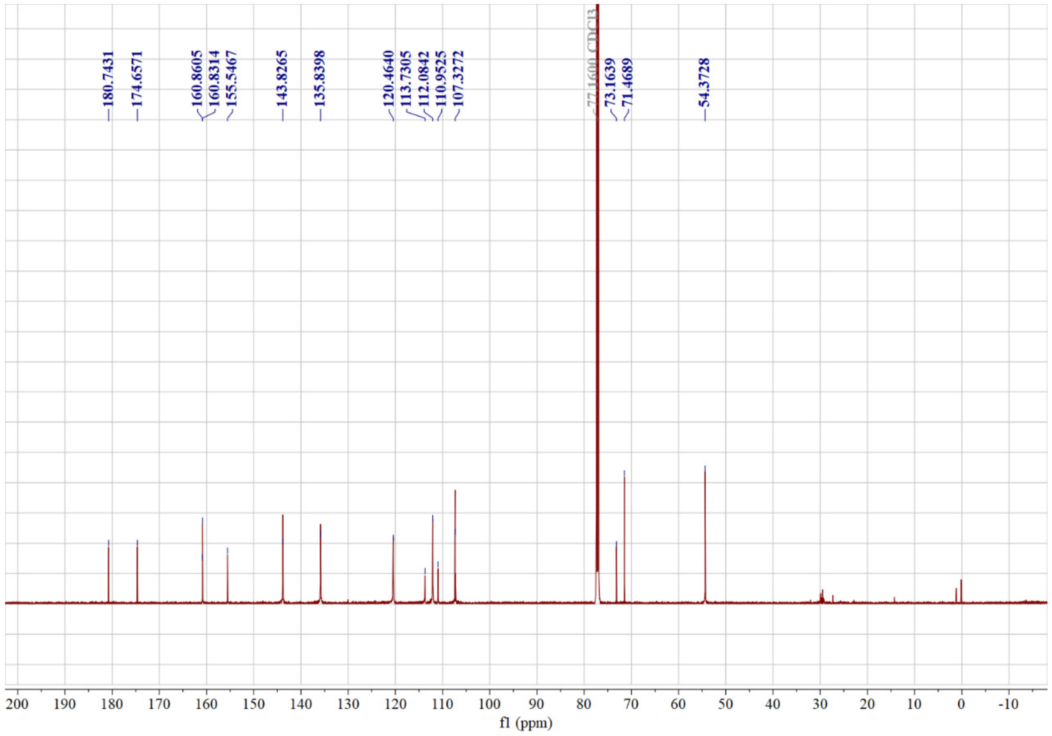

Figure S4 DEPT135 spectrum of compound **1** (150 MHz, $CDCl_3$)

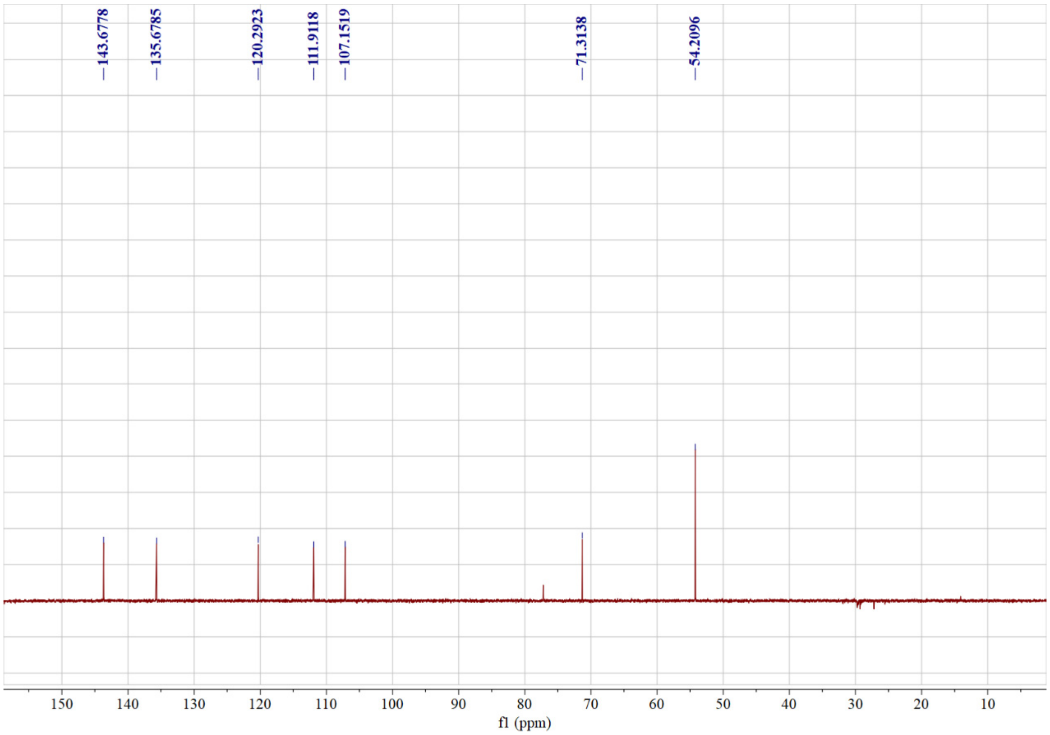

Figure S5 ^{1}H-^{1}H COSY spectrum of compound **1**

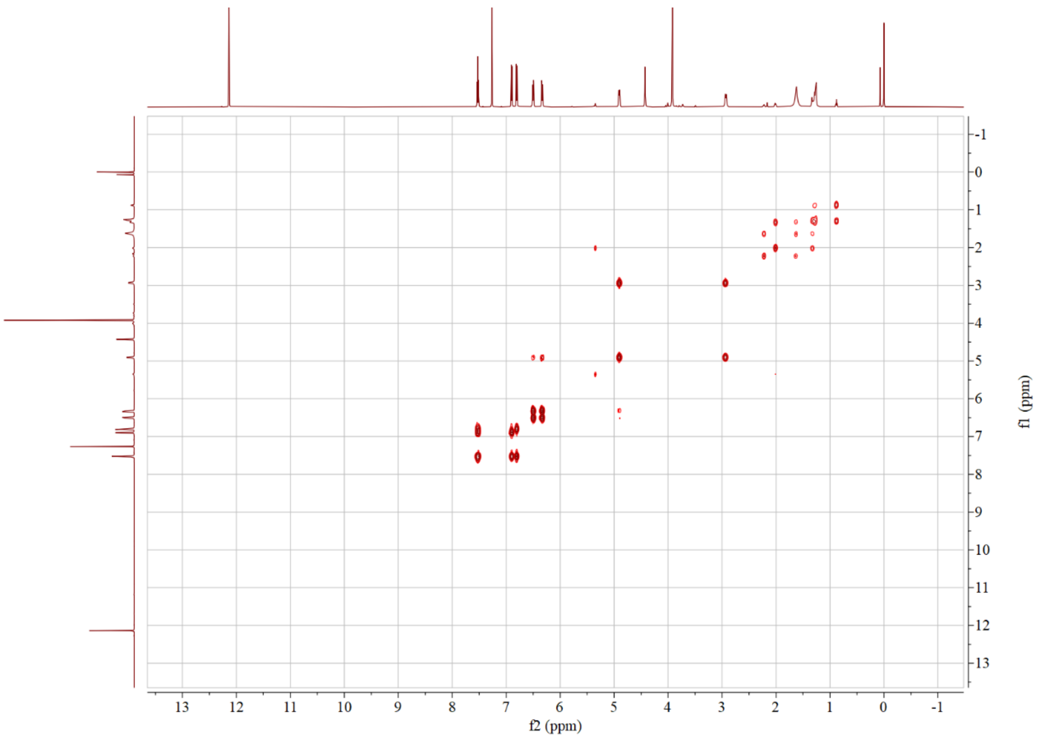

Figure S6 HSQC spectrum of compound **1**

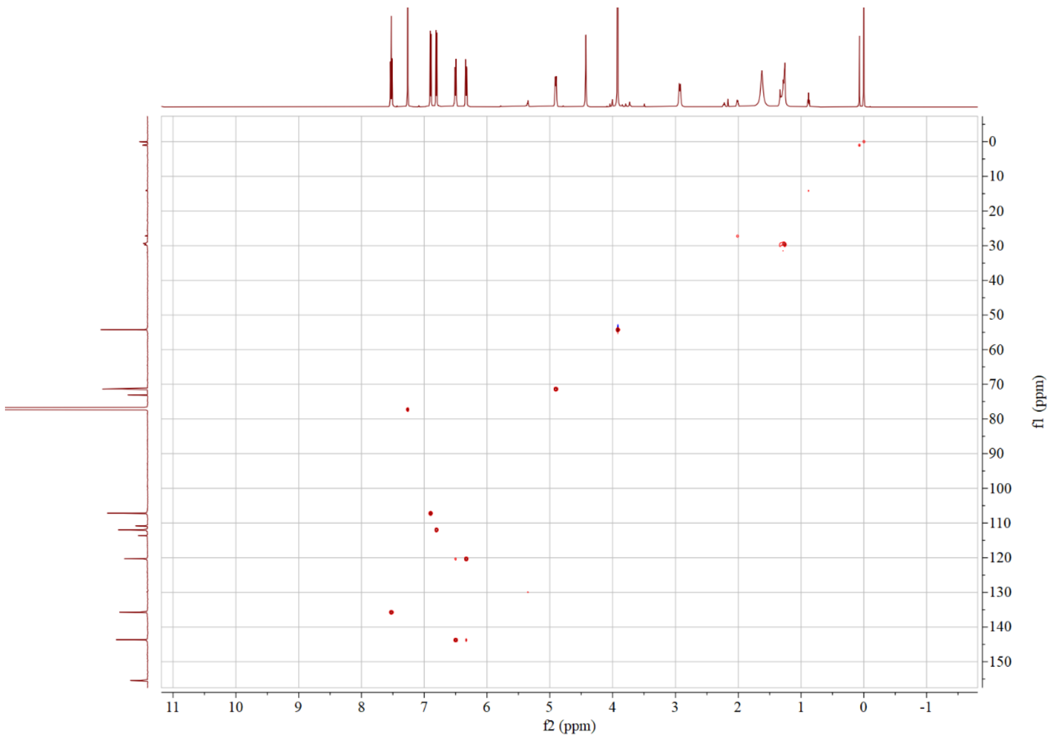

Figure S7 HMBC spectrum of compound **1**

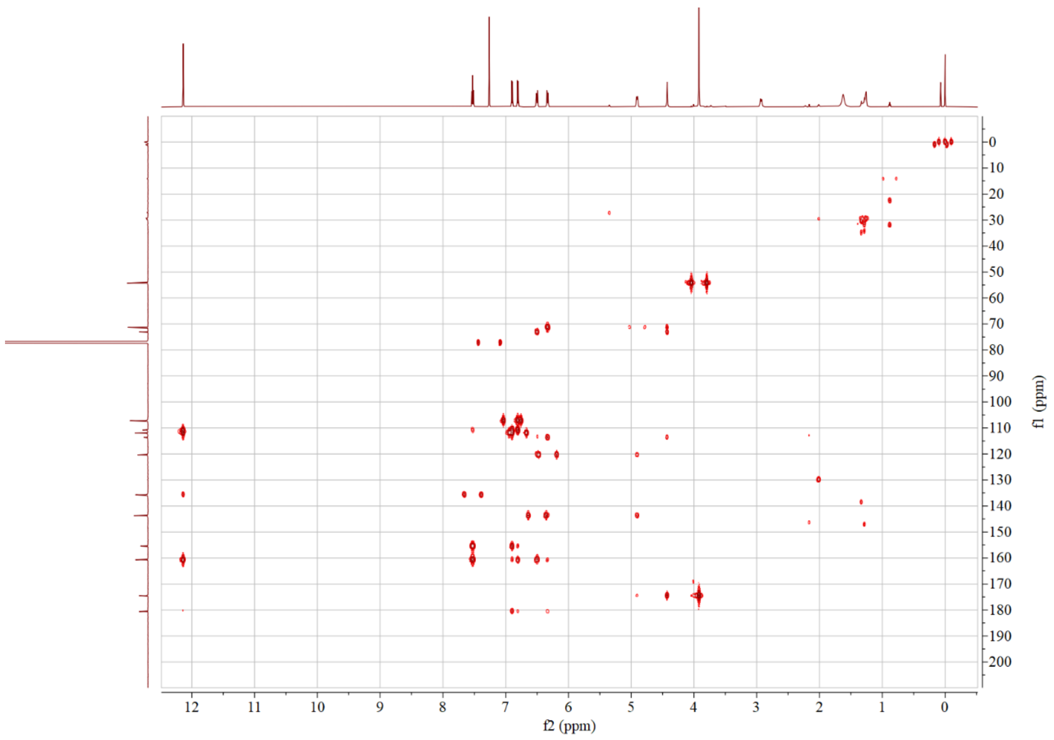

Figure S8 NOESY spectrum of compound **1**

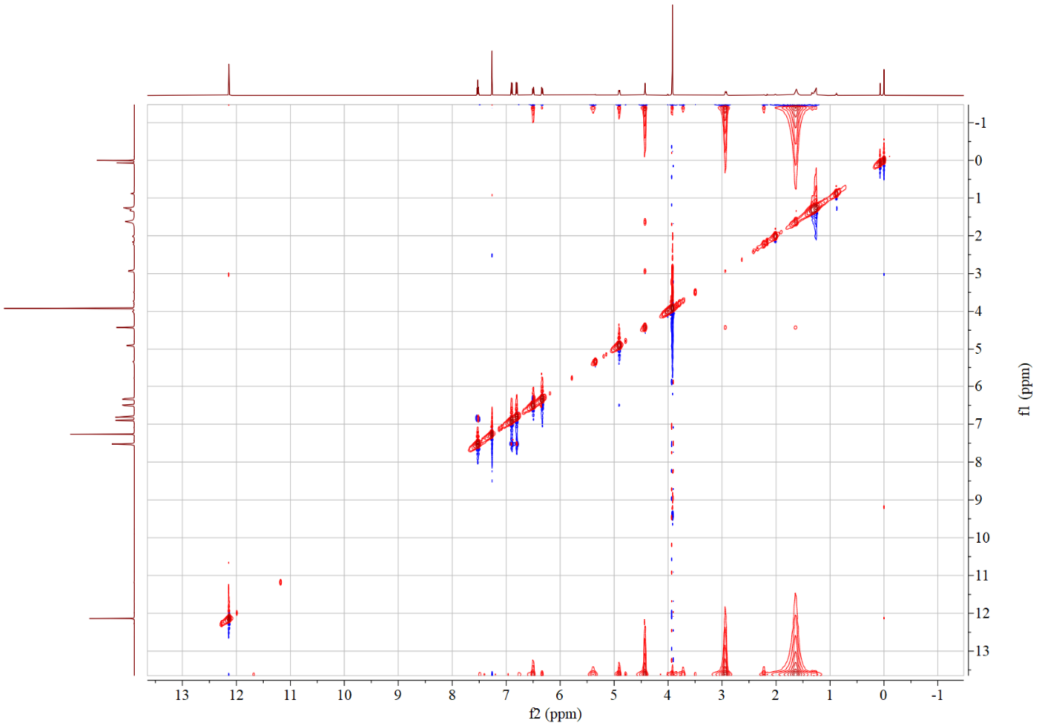

Figure S9 ^{1}H NMR spectrum of compound **2** (600 MHz, $CDCl_3$)

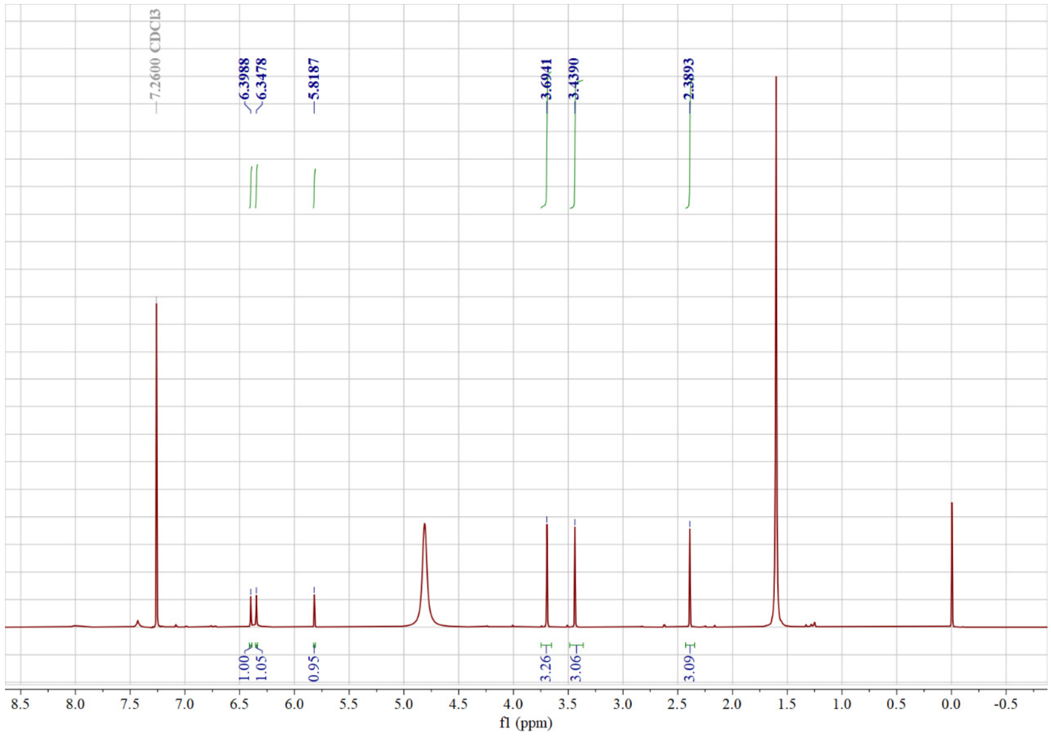

Figure S10 ^{13}C NMR spectrum of compound **2** (150 MHz, $CDCl_3$)

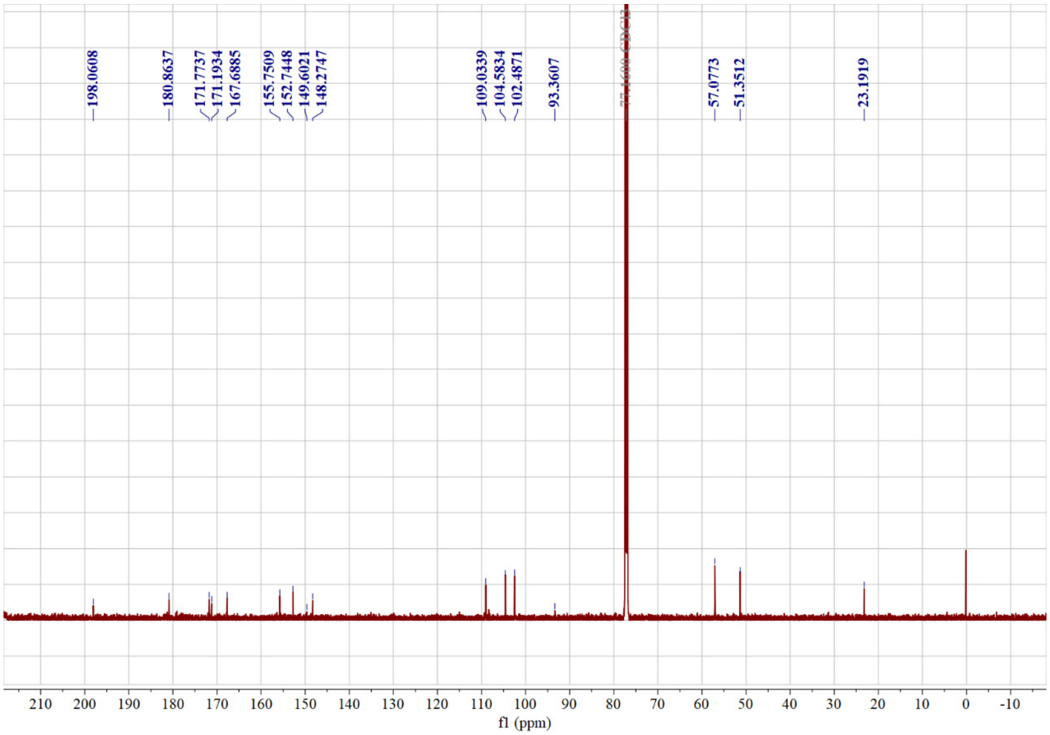

Figure S11 DEPT135 spectrum of compound **2** (150 MHz, $CDCl_3$)

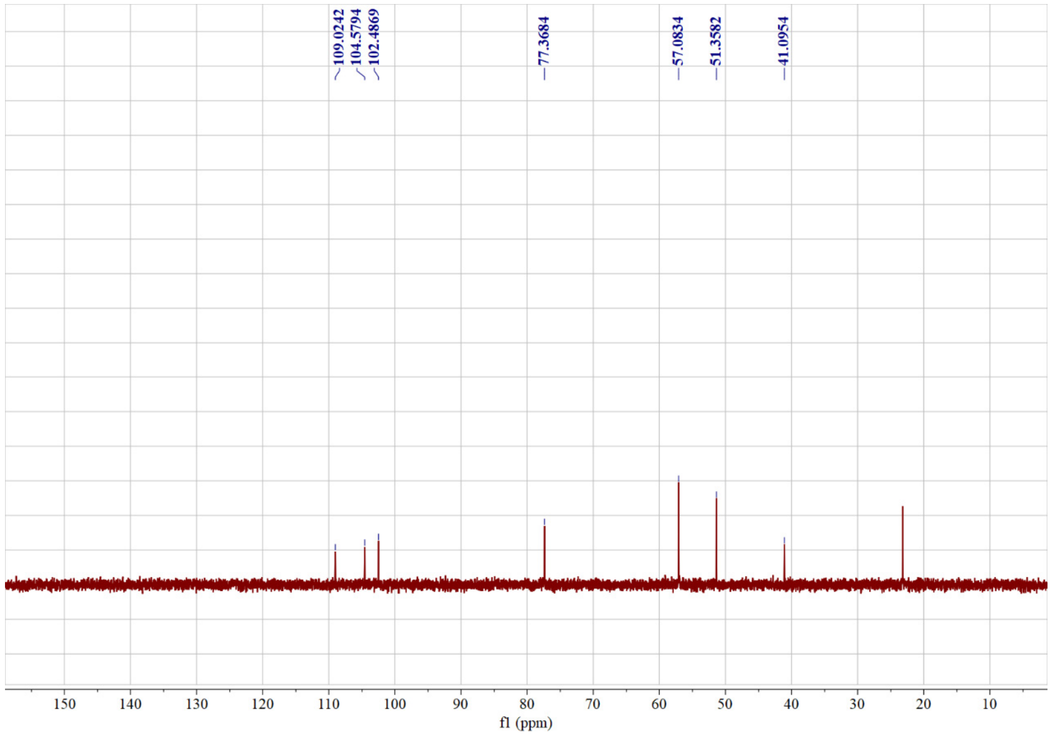

Figure S12 ^{1}H-^{1}H COSY spectrum of compound **2**

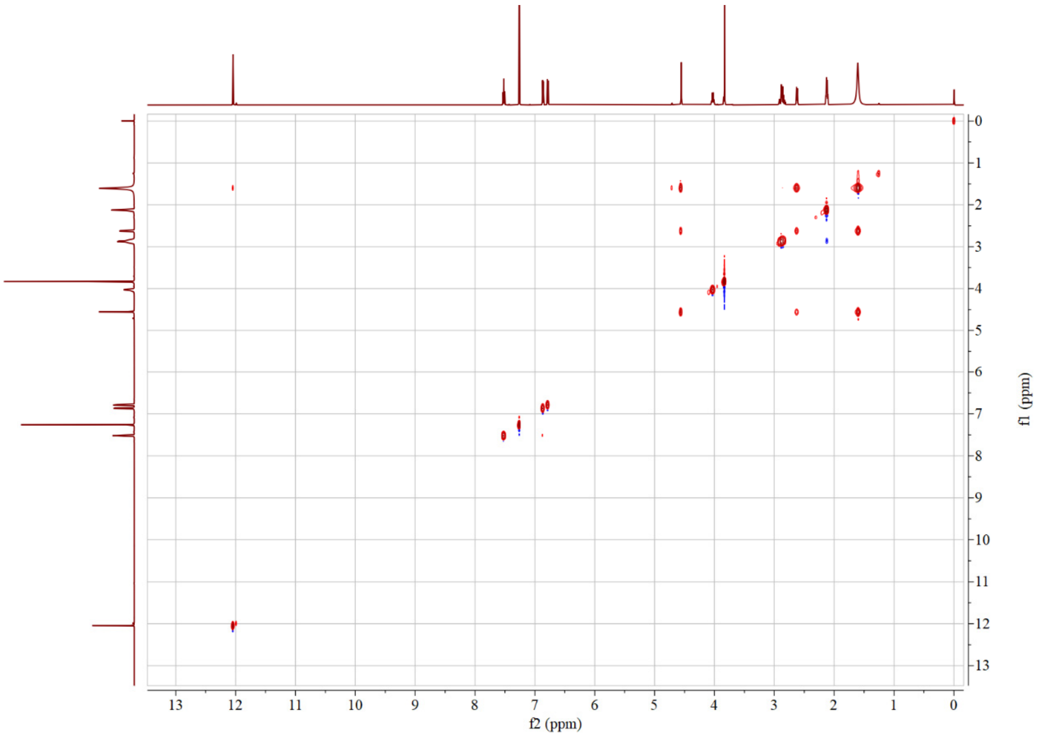

Figure S13 HSQC spectrum of compound **2**

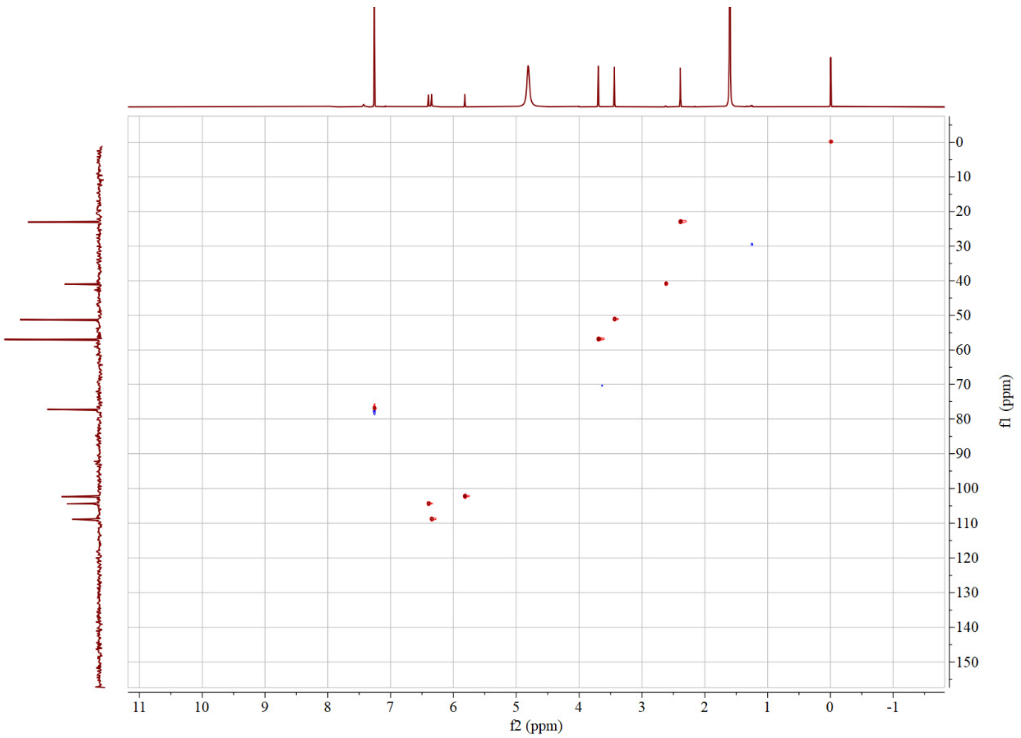

Figure S14 HMBC spectrum of compound **2**

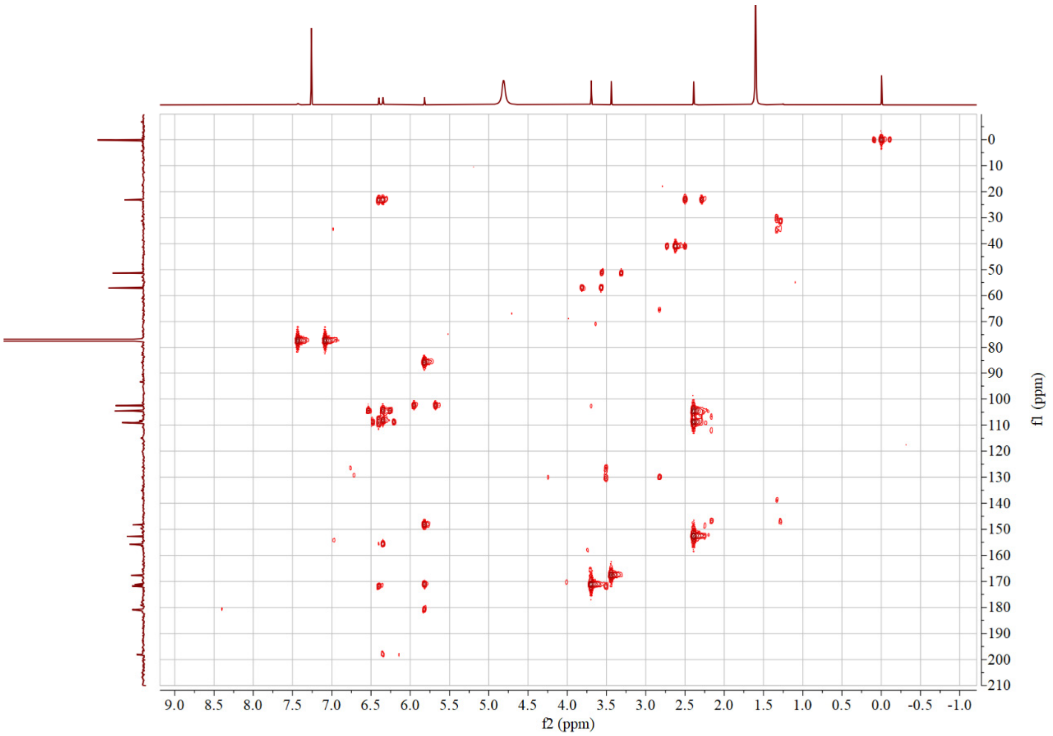

Figure S15 NOESY spectrum of compound **2**

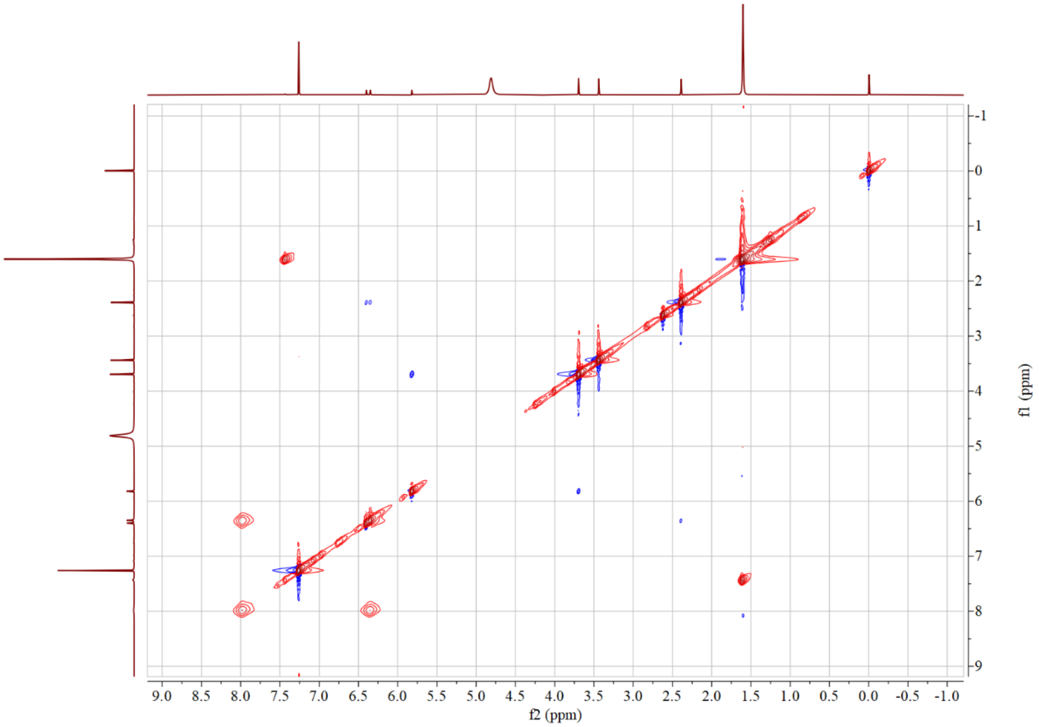

Supplement: Supplementary file 1 [file marinedrugs-19-00186-s001.pdf]
